# Healthcare service utilization patterns and patient experience in persons with spinal cord injury: a comparison across 22 countries

**DOI:** 10.1186/s12913-022-07844-3

**Published:** 2022-06-07

**Authors:** Olena Bychkovska, Piotr Tederko, Julia Patrick Engkasan, Abderrazak Hajjioui, Armin Gemperli

**Affiliations:** 1 Swiss Paraplegic Research, 6207 Nottwil, Switzerland; 2 Department of Health Sciences and Medicine, University of Lucerne, 6002 Lucerne, Switzerland; 3 Department of Rehabilitation, Medical University of Warsaw, 02637 Warsaw, Poland; 4 Department of Rehabilitation Medicine, Universiti Malaya, 50603 Kuala Lumpur, Malaysia; 5 Faculty of Medicine and Pharmacy, Sidi Mohamed Ben Abdellah University, 1975 Fes, Morocco; 6 Center for Primary and Community Care, University of Lucerne, 6002 Lucerne, Switzerland

**Keywords:** Utilization pattern, patient experience, primary healthcare, high healthcare needs, spinal cord injury, country comparison

## Abstract

**Background:**

Persons with spinal cord injury frequently visit numerous clinical settings. Such all-around experience of the system may serve as a comprehensive experience indicator. This study compared the patient experience of persons with chronic SCI in relation to healthcare service utilization patterns in 22 countries, hypothesizing that primary-care oriented patterns would offer a better experience.

**Methods:**

This study was based on International Spinal Cord Injury Survey with 12,588 participants across 22 countries worldwide. Utilization patterns/clusters were identified by cluster analysis and experience score – by the partial credit model. The association between healthcare utilization and experience at the provider and cluster level was explored by regression analysis.

**Results:**

The highest share of visits was to primary care physicians (18%) and rehabilitation physicians (16%). Utilization patterns had diverse orientations: from primary care to specialized and from inpatient to outpatient. The experience was reported as very good and good across different dimensions: 84% reported respectful treatment; 81% – clear explanations; 77% – involvement in decision making; 65% – satisfaction with care. The average experience score (0–100) was 64, highest – 74 (Brazil) and the lowest – 52 (Japan, South Korea). Service utilization at provider and at cluster levels were associated with patient experience, but no utilization pattern resulted in uniformly better patient experience.

**Conclusion:**

While there are distinct patterns between countries on how persons with chronic SCI navigate the healthcare system, we found that different utilization patterns led to similar patient experience. The observed difference in patient experience is likely determined by other contextual factors than service utilization.

**Supplementary Information:**

The online version contains supplementary material available at 10.1186/s12913-022-07844-3.

## Introduction

The design of the healthcare system and healthcare provision models play a key role in obtaining desired health outcomes [1]. It is rather a complex question of how to organize the healthcare provision, and even much so for persons with complex health conditions and high healthcare needs. The more severe the condition a person has, the less likely they are to receive comprehensive care [2], which in a vicious cycle, is leading to worsening of their health condition [3].

An example of a high needs group is persons with chronic spinal cord injury (SCI). Spinal cord injury is a rare medically complex and costly condition. The data on SCI incidence and prevalence are limited and inconsistent, hard to estimate and compare across countries [4, 5]. SCI is often accompanied by secondary conditions. Persons with SCI require multidisciplinary care [4] and tend to utilize numerous clinical settings with high frequency for follow-up treatment of acute secondary conditions [6]. In Switzerland, persons with SCI visited outpatient clinics four times and in-patient hospitals three times more frequently than the general population [7]. Because of such frequent visits to numerous settings, these patients get an all-around experience of the health system, which may serve as a comprehensive experience indicator [4].

There is little consensus on optimal provision models for this group [8], with some emphasizing the need to strengthen primary care [8, 9], while some focus on system centralization and specialized care [10]. Similarly, there is little information on how these patients use the services. From the scarce evidence available it is known that services for those who live in the community are not as developed as acute services immediately after a spinal cord injury [11]. Even though secondary conditions are often preventable and manageable in the community [12, 13], when there are few appropriate services or little expertise available, persons with SCI are instead referred to specialists or being hospitalized. Utilization data are predominantly lacking and difficult to collect, especially in low-income countries [5]. The question remains how persons with SCI living in the community utilize health care services and how different utilization models compare in terms of the patient experience.

This study aims to compare the patient experience of persons with SCI in relation to different healthcare service utilization patterns across 22 countries worldwide. Specifically, the objectives are to understand: 1) What are the healthcare service utilization patterns among persons living with chronic SCI in different countries? 2) What is the patient experience with healthcare among persons with chronic SCI in different countries? 3) Is patient experience with healthcare related to the visits of certain healthcare providers and do utilization patterns which are associated with better patient experience exist? The study hypothesis is that utilization patterns oriented on primary care are associated with better patient experience [8, 10]. Primary care was shown to ensure trust and longitudinal relationship between the doctor and the patient as well as improve system responsiveness, making the care more patient-centered [14].

## Methods

### Data collection and sampling

This study relies on the International Spinal Cord Injury (InSCI) cross-sectional, community-based, questionnaire survey conducted in 2017–2019 [5]. InSCI is the first international survey that aims to describe the lived experience of persons living with chronic SCI in the community. The survey is part of the International Learning Health System for Spinal Cord Injury Study (LHS-SCI), which is embedded in the World Health Organization’s Global Disability Plan [15]. LHS-SCI was launched in 2017 with the support of the World Health Organization (WHO), the International Society for Physical and Rehabilitation Medicine (ISPRM) and the International Spinal Cord Society (ISCoS) [5]. InSCI covers 22 countries across all WHO regions and was planned to be repeated every 5 years. The survey’s role is to gather data for further analysis leading to policy and practice changes, aimed at strengthening rehabilitation and other services both for persons with SCI and the general population [5, 16].

The study population included adults with SCI living in the community across 22 countries: Australia, Brazil, China, France, Germany, Greece, Indonesia, Italy, Japan, Lithuania, Malaysia, Morocco, the Netherlands, Norway, Poland, Romania, South Africa, South Korea, Spain, Switzerland, Thailand, and the United States (US). The study participants were adults 18 years old or older with non-traumatic or traumatic SCI. Those receiving first rehabilitation or first acute care during the data collection were excluded from the study due to the lack of experience of living with SCI in the community [17].

Each participating country had a national study center that led the 125-question questionnaire translation and adaptation, data collection and reminder management etc. A central study center (Swiss Paraplegic Research, Nottwil, Switzerland) coordinated the survey and provided recommendations on sampling as well as data collection, storage, and analysis. It evaluated and approved the sampling and data collection process in each country [17].

Based on a power analysis from the Swiss Spinal Cord Injury community survey data, a minimal target sample size of 200 participants per country was established. This minimal sample size is expected to provide sufficient power for comparative cross-country analysis. The national sampling frame contained at least 400 individuals with an expectation of a 50% response rate [5].

Details on the sample size, sampling design, participants’ recruitment strategy, survey administration mode, recruitment sources, and national study centers in each InSCI country have been thoroughly described [5, 17] (Supplementary Table 1), including in InSCI Country Reports [18]. To ensure high scientific quality standards, guidelines concerning target populations were developed along with a sampling frames hierarchy. Participating countries chose the sampling frames in the following order, arranged with descending frame representativeness: national SCI registry; databases of academic or level I trauma hospitals; databases of specialized rehabilitation institutions; databases of the patient organization or insurance agencies; samples of previous SCI studies; or a combination of these sampling frames. Since comprehensive registries on persons with SCI were lacking, countries with limited patient data and/or access to it had to draw convenience samples. Similarly, to collect the best possible data 16 out of 22 countries relied on multiple recruitment sources and all had different response mode options, including paper-pencil or online questionnaire, telephone or face-to-face interviews. Each country obtained ethical approval for conducting the survey and informed consent was signed by each study participant or participant’s authorized representative. Collected data were de-identified and stored in a secure central database [17].

Hence, from the 22 countries, eight (36%) were able to rely on predefined sampling frames: Australia, China, Germany, the Netherlands, Norway, Poland, South Africa, and Switzerland. Those countries represent 65% of the data analyzed in this particular study. While the results derived from the InSCI data might have limited representativeness, they often present the best available evidence regarding the SCI population. Results of this study should be complemented with other research before being used as a guide to practice or policy [17].

InSCI and this study in particular included participants from low- and middle-income countries, with 27% of participants being women, which represent a minority among persons with SCI. Many of the participating countries belonged to the quartile with the highest gross domestic product. Participants from lower-income settings were on average younger, with lower education, less likely to be of a foreign origin, more likely to have tetraplegia and a shorter time living with SCI [5]. Socio-demographic characteristics of each country can be found in the InSCI Cohort Profile [17].

The InSCI data allow both country and cross-country analyses. To capture both perspectives, data on the health systems and economic resources were gathered and the national and cross-country context described [19, 20]. Concerning the health system context, countries with different system types were included in this study. Most have centralized systems with medium or weak primary care strength. Overall, some countries were less described and undergo reforms, hence, their system types were less clear-cut. Australia and South Korea had the highest (87) universal healthcare index of service coverage score (an indicator of essential health services coverage, 0–100) [21] and Indonesia had the lowest (59). The percentage of the population with household expenditures on health greater than 10% of total household expenditure was the highest in China (24%), Morocco (21%) and lowest in South Africa (1%), Germany, Malaysia and Thailand (2%) (Supplementary Table 2).

### Data analysis and management

#### Healthcare experience

Patient experience is measured by four five-point Likert scale survey questions from the WHO Model Disability Survey [22]: respectful treatment; clear explanations; involvement in decision making; satisfaction with healthcare. The scale reliability coefficient (Cronbach’s alpha) of these items was 0.85. A partial credit model [23] was applied to attain an interval-scaled total experience score for each individual. The score was scaled to a 0–100 range (median 61, interquartile range 23). The partial credit model assumptions were tested by checking ordered categories via graphs; unidimensionality test though running principal component analysis (eigenvalues: 1st component = 3.1, proportion = 0.7; 2nd component = 0.6, proportion = 0.1) and factor analysis (eigenvalues: 1st factor = 2.8, proportion = 1.1; 2nd factor = 0.0, proportion = 0.0) on polychoric correlations. When testing local independence, the largest correlations were between items respectful treatment and clear explanations (0.4), and between involvement in decision making and clear explanations (0.4). Differential item functioning was tested on the following characteristics: sex, age, SCI lesion level (para- or tetraplegia), lesion type (complete or incomplete) and showed no difference.

#### Healthcare utilization

Utilization was measured by questions from the WHO Model Disability Survey [22] on visits to 12 types of health providers (dichotomous variable) and the number of hospital stays (continuous variable) in the last 12 months before completing the survey. The scale reliability coefficient (Cronbach’s alpha) of these items was 0.7. The following healthcare providers have been included in the analysis: primary care physician/general practitioner, physical and rehabilitation medicine/SCI physician, other specialist physicians, nurse/midwife, dentist, physiotherapist, chiropractor, occupational therapist, psychologist, alternative medicine specialist, pharmacist, home healthcare worker. Such categories of providers as a dietician, social worker, and the category “other provider” were excluded from the analysis. Since the survey questions about provider visits were dichotomous there was no possibility to quantify the number of times an individual visited a certain provider or to have an indicator of visit duration.

#### Association between healthcare utilization type and patient experience

To establish an association between healthcare utilization type (provider’s visit) and patient experience, a multilevel univariate and multivariable regression analysis with a country as a random effect was performed. We adjusted by the nonmodifiable socio-demographic (sex, age, migration background) and spinal cord injury characteristics (SCI severity: tetra- or paraplegia, complete or incomplete lesion; traumatic or nontraumatic etiology; years lived with injury).

#### Association between healthcare utilization pattern and patient experience

Countries’ health systems were classified with regard to healthcare service utilization among persons with SCI using an unsupervised cluster analysis. Cluster analysis was based on utilization indicators described above: visits to 12 types of health providers and the number of hospital stays in the last 12 months before completing the survey. For each country, the relative share in a percentage of visits to each provider, or of no visit to any provider, were computed, as well as the percentage of individuals with no, one, two, or more than three inpatient hospitalizations. Hierarchical cluster analysis was conducted on a dissimilarity matrix based on Gower distance and Ward’s method linkage as a final procedure. Several other types of cluster analyses on other linkages (single-linkage, complete-linkage, average-linkage, and centroid-linkage) along with k-means clustering with 5-10 k were as well performed to verify the results and country memberships. The cluster analysis groups countries in a way that inside each cluster the countries are not identical, but still are more similar to each other than to others. Those systems that are too distinct from all other ones are left alone in a cluster.

The association of patient experience score as a dependent variable with utilization cluster as an independent variable was explored using univariate and multivariable regression analysis. We adjusted by the nonmodifiable socio-demographic (sex, age, migration background) and spinal cord injury characteristics (SCI severity: tetra- or paraplegia, complete or incomplete lesion; traumatic or nontraumatic etiology; years lived with injury).

The study was based on a predefined data analysis protocol, which was approved by the InSCI Committee before the study start. All statistical analyses were conducted using Stata 16.

## Results

### Socio-demographic characteristics of study participants

The survey was conducted among 12,588 participants, as three participants had to be deleted from the study after a data quality check. The response rates were only available for countries with predefined sampling frames and are the following: South Africa 54%, Norway 42%, Switzerland 39%, the Netherlands 33%, Germany 32%, Poland 32%, Australia 27%, China 23% [17]. The sample was predominantly male (73%), with an average age of 51 years, mostly without a migrant background (91%), and living with others (77%). The majority of the participants had paraplegia (61%) for 13 years on average with an incomplete lesion (60%) and traumatic etiology (80%) (Table 2). Additionally, socio-demographic characteristics by country were described by Fekete et al. (2020) [17].

### Healthcare utilization

The healthcare providers with the highest share of visits in the last 12 months were primary care physicians (share of 18% among all healthcare providers) and physical and rehabilitation medicine (PRM)/SCI physicians (16%), followed by other specialist physicians (11%) and physiotherapist (13%) (Table 1). The chiropractor had the smallest share (1%) among the 12 providers. Across all countries, 26% of patients visited only one healthcare provider. Two, three, four, five, six, or seven providers were visited by 13, 13, 12, 11, 9, and 6% of individuals, respectively. Less than 5% visited eight or more providers. A third of the participants (34%) did not visit any healthcare provider in the last 12 months. More than half of respondents (54%) did not have any inpatient stays in the last 12 months, while 19% had one stay, 9% had two and another 9% had three or more stays.

**Table 1:** Utilization of healthcare services during 12 months

			Clusters														
			1		2		3		4	5		6		7	8		9
	Total		AU, ZA, US^a^		JP, KR^a^		CH, DE, LT, NL, NO^a^		CN^a^	FR, GR, IT, MA, ES^a^		ID, PL^a^		BR^a^	MY, TH^a^		RO^a^
	Mean	Min/Max	Mean	Min/Max	Mean	Min/Max	Mean	Min/Max	Mean	Mean	Min/Max	Mean	Min/Max	Mean	Mean	Min/Max	Mean
*No visits to any provider, %*	3.9	0.1/ 33.6	1.6	0.5/3.1	3.0	0.7/5.3	0.5	0.2/1.0	33.6	2.3	0.1/5.2	10.0	8.4/11.7	0.3	2.5	1.0/3.9	0.9
*Relative share of providers among all providers, %*																	
Primary care physician / GP	17.8	8.5/31.4	17.3	16.3/18.8	9.4	8.5/10.3	22.3	17.4/31.4	12.0	17.5	12.2/23.7	25.5	23.5/27.5	17.4	11.2	9.8/12.5	18.8
PRM^b^ / SCI physician	15.5	7.0/28.2	12.5	8.1/16.5	22.6	17.6/27.6	8.6	7.0/10.1	23.1	16.0	13.4/17.9	9.8	7.1/12.4	19.3	27.9	27.7/28.2	16.7
Other specialist physician	11.3	4.0/22.2	8.9	5.6/11.8	14.1	12.3/15.9	12.1	11.5/12.9	4.0	13.4	9.4/22.2	13.3	10.4/16.2	6.2	8.1	6.4/9.9	12.2
Nurse of midwife	6.8	1.2 /12.5	7.5	5.4/9.5	7.6	3.5/11.7	3.8	1.2/4.6	5.7	7.0	4.7/9.3	11.6	10.7/12.5	7.8	9.2	7.0/11.5	3.8
Dentist	8.4	0.6/16.6	8.6	4.8/11.8	9.1	7.6/10.6	14.8	12.9/16.6	0.6	7.5	4.2/9.9	5.2	2.9/7.5	5.6	4.3	3.8/4.8	4.9
Physiotherapist	12.9	6.6/19.3	11.3	10.1/12.2	11.8	11.2/12.4	14.1	11.0/16.2	6.6	13.0	10.3/15.2	10.8	7.8/13.8	19.3	13.3	13.3/13.3	17.2
Chiropractor	0.7	0.0/2.8	1.5	0.2/2.5	0.7	0.6/0.7	0.6	0.0/1.2	2.8	0.8	0.2/1.2	0.0	0.0/0.0	0.0	0.1	0.1/0.1	0.0
Occupational therapist	4.6	0.0/10.5	7.4	5.6/8.8	5.4	4.2/6.6	5.0	3.3/6.7	0.0	1.3	0.9/1.8	1.5	1.2/1.8	10.5	8.3	8.1/8.5	6.8
Psychologist	2.8	0.2/10.6	3.1	2.7/3.8	0.7	0.4/1.1	2.6	2.1/3.2	0.2	2.7	1.6/13.7	3.4	2.5/4.3	10.6	1.9	1.9/1.9	2.7
Alternative therapist	2.4	0.8/6.6	2.0	1.3/2.6	2.2	1.6/2.9	2.7	1.6/3.3	3.4	1.8	1.0/3.8	2.3	0.8/3.8	0.9	5.1	3.6/6.6	1.5
Pharmacist	8.9	0.4/15.0	11.6	9.6/12.6	7.7	7.5/8.0	7.5	1.4/15.0	7.9	13.4	10.2/15.0	4.1	3.8/4.5	0.4	5.9	3.1/8.7	12.8
Home healthcare worker	4.0	0.0/7.8	6.8	6.0/7.5	5.9	5.5/6.3	5.4	2.6/7.8	0.0	3.3	0.6/4.6	2.8	1.2/4.4	1.7	2.2	1.2/3.1	1.7
*Number of inpatient stays*^*c*^*, %*																	
0 stays	53.7	23.2/74.8	54.9	50.1/58.0	51.8	49.0/54.6	55.2	38.8/68.5	65.0	57.9	41.7/74.8	53.8	51.9/55.7	53.7	49.2	45.3/53.1	23.2
1 stay	19.3	11.5/33.3	15.5	11.5/19.3	16.7	15.6/17.9	19.5	13.5/26.9	19.0	18.4	13.6/25.2	20.1	18.4/21.8	33.3	23.1	17.1/29.1	16.7
2 stays	8.9	4.3/27.8	9.2	6.0/11.8	5.3	4.9/5.6	7.3	4.3/9.7	6.7	8.2	5.8/10.3	9.8	9.5/10.2	9.5	8.1	7.8/8.4	27.8
3 and more stays	8.9	0.5/30.1	13.6	10.8/18.0	7.6	6.6/8.7	7.6	2.6/12.5	9.2	5.4	0.5/12.4	6.6	4.5/8.8	3.5	9.6	7.2/12.1	30.1

^a^ AU – Australia, BR – Brazil, CN – China, FR – France, DE – Germany, GR – Greece, ID – Indonesia, IT – Italy, JP – Japan, LT – Lithuania, MY – Malaysia, MA – Morocco, NL – the Netherlands, NO – Norway, PL – Poland, RO – Romania, ZA – South Africa, KR – South Korea, ES – Spain, CH – Switzerland, TH – Thailand, US – the United States

^b^ PRM – physical and rehabilitation medicine

^c^ Missing values: total: 9.2%, (Cluster) 1: 6.8%, 2: 18.6%, 3: 10.4%, 4: 0.1%, 5: 10.1%, 6: 9.7%, 7: 0%, 8: 10%, 9: 2.2%

### Characteristics of healthcare utilization clusters

Nine service utilization clusters were identified by the performed unsupervised cluster analysis (Table 1). Cluster 4 (China) had the lowest service visits across many services, while Cluster 7 (Brazil) featured the highest and most diverse use of services. Below provided are the description of the utilization clusters.

*Cluster 1 (Australia, South Africa, US), characterized by many visits and almost equal reliance on primary and specialized care.* In this cluster general practitioner (GP) services were used slightly more than PRM physician/SCI specialist services. Home workers, chiropractors, or occupational therapists were frequently visited. Inpatient stays were slightly higher than the overall average.

*Cluster 2 (Japan, South Korea), characterized by a strong reliance on SCI specialized outpatient care*. These countries have the bigger share of visits to a PRM physician/SCI specialist (23%), and to other specialized physicians (14%) than to a GP (9%). Dentists were slightly more visited compared to other clusters (9%), while visits to psychologists were among the lowest (1%). A majority (52%) of patients in this cluster had no inpatient stays.

*Cluster 3 (Switzerland, Germany, Lithuania, Norway, the Netherlands), characterized by a primary care-oriented system with almost equal use of specialized services.* This cluster had the second highest share of GP visits (22%) after Cluster 6 (Indonesia, Poland) (26%). The use of PRM physician/SCI specialist services (9%) was lower than the use of GPs (22%), yet other specialist physicians were often visited (12%). The percentage of dentist visits was the highest among all clusters (15%), while the use of nursing services was the lowest (4%).

*Cluster 4 (China), characterized by low healthcare service utilization and reliance mostly on SCI specialized outpatient care, with some consideration of complementary and alternative medicine.* This cluster had the largest percentage of those that indicated not visiting any healthcare provider (34%). Similar to Cluster 2 (Japan, South Korea), this cluster had an almost twice as large share of visits to a SCI specialist (23%) than to a GP (12%). Low attendance across multiple services was found: dentist (1%), home healthcare worker (0%), psychologist (0%), and occupational therapist (0%). On the other hand, the share of visits to the chiropractor (3%) and alternative medicine specialist (3%) was the largest among all clusters.

*Cluster 5 (France, Greece, Italy, Morocco, Spain), characterized by a similar use of primary and specialized services and a moderate number of inpatient stays.* The patients in these countries had almost equal (18% vs. 16%) shares of visits to GP and PRM specialists. In addition, the attendance of occupational therapist was among the lowest (1%) and pharmacist services (13%) was higher than in other clusters.

*Cluster 6 (Indonesia, Poland), characterized by a primary care-oriented system with infrequent visits.* Among the countries in this cluster, the number of persons with no visits to any healthcare provider was almost three times higher than the average across all clusters (10%). GP services had the highest share across all clusters (26%), along with nurse or midwife services (12%). Inpatient stays among these countries were similar to the overall cluster average.

*Cluster 7 (Brazil), characterized by a generally specialized system with frequent visits and hospital stays.* This cluster had the lowest percentage of persons without any visit to a healthcare provider (0.3%). For example, the share of visits to the PRM physician was high (19%) and visits to a physiotherapist was the highest among all clusters (19%). Individuals with SCI in this cluster frequently used diverse services such as an occupational therapist, chiropractor, physiotherapist, and psychologist. One-third of respondents had one hospital stay and here was the lowest percentage of three or more hospital stays among all clusters (4%).

*Cluster 8 (Malaysia, Thailand), characterized by a inpatient-oriented SCI specialized system.* In this cluster, the share of SCI specialist visits (28%) was twice as large as those to the GP (11%). Hospitalizations were above the cluster average. Alternative medical specialist services were frequently used (5%) while pharmacist services were the least used among all clusters (6%).

*Cluster 9 (Romania), characterized by a inpatient-oriented care system with the highest number of hospitalizations*. This cluster had the lowest number of persons without any hospital stay (23%), almost half of the share less than the cluster with the second-lowest stays (Cluster 8). The percentage of persons with two (28%) and three or more (30%) hospital stays was almost three times higher than the overall cluster average. Unlike other clusters, the percentage of visits to GP, PRM physician/SCI specialist, and physiotherapist was similar (19, 17, and 17%). Services of pharmacists (13%) and occupational therapists (7%) were frequently utilized.

### Socio-demographic characteristics of the healthcare utilization clusters

The highest percentage of males was in Cluster 6 (Indonesia, Poland) (80%) and 7 (Brazil) (79%). The mean age was the lowest in Cluster 9 (Romania) (38 years) and highest in Cluster 3 (Switzerland, Germany, Lithuania, Norway, the Netherlands) (56 years). The percentage of respondents with an immigrant background was below 1% in most clusters, with larger shares in Cluster 1 (Australia, South Africa, US) (19%), Cluster 3 (Switzerland, Germany, Lithuania, Norway, the Netherlands) (11%), and Cluster 5 (France, Greece, Italy, Morocco, Spain) (6%). The percentage of participants with tetraplegia was highest in Cluster 2 (Japan, South Korea) (42%), followed by Clusters 7 (Brazil) (40%) and 6 (China) (40%). In other clusters, this percentage was between 31 and 38%. Cluster 4 (China) and Cluster 7 (Brazil) had the highest percentage of those with an incomplete lesion, 75 and 79% respectively. This percentage was lowest in Cluster 2 (Japan, South Korea) (40%). The percentage of those with nontraumatic etiology was highest (32 and 30%) in Cluster 4 (China) and Cluster 7 (Brazil), and lowest in Cluster 2 (Japan, South Korea) (8%).

### Patient experience

The majority of the respondents rated their healthcare experience as good or very good across all countries and all four experience categories: respectful treatment 84%; clear explanations 81%; involvement in decision making 77%; satisfaction with healthcare 65%. A small fraction of responders rated their healthcare experience as bad (respectful treatment 3%; clear explanations 4%; involvement in decision making 4%; satisfaction with healthcare 9%) or very bad (respectful treatment 1%; clear explanations 1%; involvement in decision making 2%; satisfaction with healthcare 3%). In terms of experience (total score (0–100)) by country, the lowest scores were attained by Morocco (44), followed by South Korea (49), Lithuania (55), China (55), Poland (57) and Italy (57). The highest country experience scores were observed in the US (78), Spain (77), Brazil (74), Australia (73), Malaysia (72), and Switzerland (71).

The average experience score across all healthcare utilization clusters was 64 (Table 2). The highest cluster score was 74 in Cluster 7 (Brazil) and the lowest was 52 in Cluster 2 (Japan, South Korea). There was a wide variability of experience scores within the clusters: the difference among the individual country’s experience scores was 33 points between countries in Cluster 5 (France, Greece, Italy, Morocco, Spain), 16 in Cluster 3 (Switzerland, Germany, Lithuania, Norway, the Netherlands), 13 in cluster 1 (Australia, South Africa, US), 12 in Cluster 2 (Japan, South Korea), 5 in Cluster 6 (Indonesia, Poland), and 3 in Cluster 8 (Malaysia, Thailand).

**Table 2:** Characteristics of healthcare utilization clusters

*Clusters*		1	2	3	4	5	6	7	8	9
*Countries*	Total	AU, ZA, US^a^	JP, KR^a^	CH, DE, LT, NL, NO^a^	CN^a^	FR, GR, IT, MA, ES^a^	ID, PL^a^	BR^a^	MY, TH^a^	RO^a^
*Experience score, 0-100*^*b*^ – mean	64	72 (AU: 73, ZA: 65, US: 78)	52 (JP: 61, KP: 49)	67 (CH: 71, DE: 64, LT: 55, NL: 70, NO: 69)	55	62 (FR: 70, GR: 58, IT: 57, MA: 44, ES: 77)	58 (ID: 62, PL: 57)	74	70 (MY: 72, TH: 69)	60
*Socio-demographic characteristics*										
Male – % (min, max)	72.8 (58.1, 83.1)	71.9 (58.1, 74.9)	77.2 (75.6, 82.1)	70.1 (62.8, 71.2)	71.1	72.1 (69.8, 74.3)	80.4 (67.2, 83.1)	79.1	74.9 (70.9, 79.2)	72.2
Age – mean (min, max)	51.3 (38.2, 58.6)	54.3 (38.4, 57.5)	49.7 (48.0, 54.8)	55.8 (42.3, 58.6)	49.7	47.8 (38.6, 51.6)	46.3 (43.8, 46.8)	44.2	42.6 (40.1, 45.0)	38.2
Migrant background – % (min, max)	7.6 (0.0, 22.8)	19.3 (3.5, 22.8)	0.3 (0.2, 0.3)	10.9 (0.9, 18.4)	0.0	5.8 (0.5, 12.5)	0.6 (0.0, 0.7)	0.0	0.6 (0.3, 1.0)	0.5
Living situation – % (min, max)										
Alone	18.3 (4.1, 31.0)	20.9 (6.5, 22.9)	24.8 (15.6, 27.9)	24.7 (12.4, 28.5)	5.3	17.8 (4.4, 31.0)	11.0 (5.0, 12.3)	6.5	4.5 (4.1, 5.0)	10.6
With others	76.8 (65.1, 94.8)	73.1 (63.3, 80.3)	72.3 (69.0, 82.1)	69.4 (65.9, 87.2)	91.9	79.9 (65.1, 94.8)	82.1 (77.6, 83.0)	92.5	88.0 (87.2, 88.8)	88.9
In institution	3.3 (0.0, 27.1)	5.6 (0.5, 27.1)	1.2 (1.0, 1.7)	3.0 (0, 3.4)	2.4	1.4 (0.5, 1.9)	6.0 (3.9, 15.9)	0.0	6.0 (4.7, 7.2)	0.5
*SCI characteristics*										
Tetraplegia – % (min, max)	36.4 (10.0, 49.0)	37.0 (36.7, 38.4)	41.7 (39.3, 49.0)	38.2 (29.8, 46.9)	33.1	31.1 (25.7, 36.0)	39.6 (10.0, 45.7)	40.3	27.5 (25.6, 29.5)	30.6
Incomplete lesion – % (min, max)	60.4 (25.2, 79.1)	64.6 (46.7, 71.4)	40.3 (35.8, 41.8)	62.1 (25.2, 79.0)	74.5	55.8 (53.5, 60.7)	54.7 (54.1, 57.7)	79.1	56.8 (54.7, 59.1)	67.1
Nontraumatic etiology – % (min, max)	19.0 (0.0, 36.5)	13.8 (0.0, 16.3)	8.3 (7.8, 9.9)	22.0 (6.4, 36.5)	32.3	21.4 (14.0, 29.6)	10.9 (10.7, 11.9)	29.9	14.1 (13.8, 14.4)	16.2
Years lived with SCI – mean (min, max)	13.2 (3.3, 20.0)	16.0 (10.3, 17.2)	16.7 (15.6, 20.0)	14.8 (8.4, 19.1)	4.5	13.8 (7.0, 18.1)	13.3 (11.0, 13.8)	3.3	8.7 (8.3, 9.2)	8.2

^a^ AU – Australia, BR – Brazil, CN – China, FR – France, DE – Germany, GR – Greece, ID – Indonesia, IT – Italy, JP – Japan, LT – Lithuania, MY – Malaysia, MA – Morocco, NL – the Netherlands, NO – Norway, PL – Poland, RO – Romania, ZA – South Africa, KR – South Korea, ES – Spain, CH – Switzerland, TH – Thailand, US – the United States

^b^ Healthcare experience score obtained after Rasch analysis

### Association between healthcare utilization and patient experience

Utilization of specific healthcare providers was associated with patient experience on an individual level (Supplementary Table 3). In both univariate (*N* = 9818, chi-square = 1563, *p*-value < 0.001) and multivariable (*N* = 9423, chi-square = 1386, *p*-value < 0.001) models the providers that were associated with higher experience score were: physical and rehabilitation medicine (PRM)/SCI physician and dentist, while with lower experience score: nurse/midwife, psychologist, alternative medicine specialist and homecare worker. The number of inpatient stays was not associated with healthcare experience. The regression coefficients did not significantly change between the models. Living with tetraplegia was associated with having 1 point lower experience score than living with paraplegia. Those with incomplete lesion had a 1 point higher experience score than those with a complete lesion. Other healthcare providers and characteristics such as sex, age, migration background, injury etiology, and time since injury showed no association with experience score on an individual level. Country explained 18% of the total variance in both univariate and multivariable models.

**Fig. 1:**
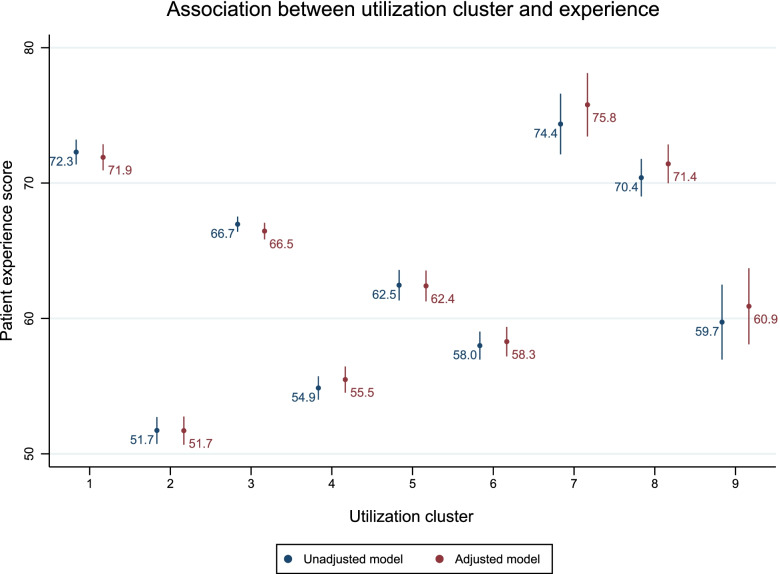
Association between utilization cluster and experience. Patient experience score range: 0–100. Cluster numbers and countries: 1 – Australia, South Africa, the United States; 2 – Japan, South Korea; 3 – Switzerland, Germany, Lithuania, the Netherlands, Norway; 4 – China; 5 – France, Greece, Italy, Morocco, Spain; 6 – Indonesia, Poland; 7 – Brazil; 8 – Malaysia, Thailand; 9 – Romania

Utilization pattern was associated with patient experience on a cluster level by univariate (*N* = 12,588, R^2^ = 0.11) and multivariable (*N* = 11,838, R^2^ = 0.12) regression analysis. (Supplementary Table 4; Fig. 1) The associations did not significantly differ when adjusting for socio-demographic and SCI lesion characteristics. Persons of age 46–60 years and those older than 76 had an experience score higher by 2 points than those younger than 30 years, while for persons aged 61–75 years this score was 4 points higher. Living with tetraplegia was associated with having a 1 point lower experience score than living with paraplegia. Those with incomplete lesion had a 2 points higher experience score than those with a complete lesion. Other characteristics such as sex, migration background, injury etiology, and time since injury showed no association with experience score.

## Discussion

This study compared the patient experience of persons with chronic SCI in relation to different healthcare service utilization patterns. Nine clusters of utilization patterns were identified, which reflect how follow-up healthcare services were actually used. While there is a difference in patient experience among these utilization clusters, contradictory to our hypothesis, neither pattern led to a uniformly superior patient experience. The specific aspects of patient experience that this study measured (decision involvement, respectful treatment, clear explanations, satisfaction) seem to be achieved by patterns with different care orientation alike.

Regarding the healthcare utilization patterns, our finding that persons with chronic spinal cord injury frequently use various follow-up healthcare services across different healthcare settings is supported by the literature [6, 8, 24, 25]. Although persons with complex health conditions have greater difficulties accessing care in general, including specialized care and rehabilitation [24], those with SCI have a higher likelihood of doctor visits [6]. These findings are particularly evident in certain countries with high frequency of use of different services (e.g., Australia, South Africa, US (Cluster 1); Brazil (Cluster 7)) and high frequency of inpatient stays (e.g., Malaysia and Thailand (Cluster 8); Romania (Cluster 9)). Even in clusters with countries with strong and medium primary care strength (Supplementary Table 2) the visits and hospitalizations are comparable to countries with weak strength.

As for the association between the utilization cluster and the patient experience, it was found that different utilization patterns led to similar experience score. Hence, longitudinal relationship and trust between the healthcare provider and the patient, which is shown to be established in primary care [14], in persons with SCI might also be equally associated with specialized care. Similarly to the literature, our findings on utilization suggest that often persons with a complex condition have to use complementary specialist services [26]. Especially the SCI management is often being redirected to specialized care, emergency and inpatient stays [8, 11, 27]. This could mean that gatekeeping may not be functioning for those with a complex medical conditions, such as SCI [9, 28]. While the gatekeeping’s role of primary care is to assure the right allocation of resources with cost containment and improved health [28], it remains unclear if this role is being well-performed in SCI care. Primary care services may not be prepared enough for persons with SCI, while emergency and inpatient stays are not ideal for prevention and follow-up management of preventable conditions [29].

Visits to the GP were not associated with better or worse patient experience. The number of inpatient stays was as well not associated with patient experience. Visits to other providers were associated with experience, however, with small effect sizes of maximum 3 points higher or lower. Hence, the difference in observed patient experience between countries or clusters of countries cannot be explained by the utilization of different types of healthcare providers or their accessibility. Likely, other contextual factors might play a role in shaping patient experience.

Although it has been established that healthcare in high-income countries is often better performing than in low-income countries [4, 24, 30], this was not the case in our study for persons with chronic SCI in terms of the patient experience. For example, Brazil (Cluster 7) and Malaysia (Cluster 8) showed one of the highest patient experience scores, while Japan and South Korea (Cluster 2) had one of the lowest. The difference may also stem from the fact that in this study we focus on a certain indicator, namely patient experience, based on healthcare provider visits. In addition, despite healthcare systems generally being viewed as one single structure and equated with the country’s boundaries, healthcare may in reality be fragmented, differing geographically and personally [29, 31], and this study does not allow for representativeness of the whole country. We found that the country as well as utilization pattern have a limited influence on the healthcare experience. The variance of 18% was found on the country level, including the effect of the countries’ healthcare systems. On a cluster level, the variance explained by the utilization pattern was 11–12% in both models, hence, other factors also play a role in explaining experience, some of which we have adjusted for.

This study had some limitations. Firstly, the sampling setting and strategy may have affected the identified utilization types. In certain participating countries, the sampling setting was limited to rehabilitation facilities (Brazil, Germany, the Netherlands, Norway), acute or general hospitals (China, Spain). This selection may have resulted in more specialized care oriented utilization types. Additionally, the sampling frames in most countries covered only a certain region and do not represent the entire country [17]. It remains unclear if certain countries showed higher use of specialized services because of such overall system orientation for the entire population, with specifically persons with SCI in those countries directed to specialists [9], or due to our survey sampling setting oriented on specialized care in those countries [17]. Secondly, to reach the respondents and adapt to their possibilities in completing the survey, the data collection methods (e.g. interview vs. survey) altered among participating countries [17] (Supplementary Table 1). This could have led to a potential bias and difference in data quality. Overall, the experience was found to be good on average across all clusters and countries, and that also might be because those with worse experience were harder to reach and recruit for the study. Thirdly, we only controlled for the nonmodifiable factors in the regression analysis, while there could be other potential factors influencing patient experience. The results of this study should be complemented with other research before being used as a guide to practice or policy.

## Conclusion

While there are distinct patterns between countries on how persons with chronic SCI navigate the healthcare system, we found that different utilization patterns led to similar patient experience. The observed difference in patient experience in clusters or countries is likely determined by other contextual factors than how healthcare services were used.

## Supplementary Information

**Additional file 1: Supplementary Table 1.** InSCI study design characteristics by country.

**Additional file 2: Supplementary Table 2.** Characteristics of the healthcare systems of InSCI countries.

**Additional file 3: Supplementary Table 3.** Association between healthcare utilization type and patient experience.  

**Additional file 4: Supplementary Table 4.** Association between healthcare utilization pattern and patient experience.

## Data Availability

The data that support the findings of this study are available from the InSCI Study Group but restrictions apply to the availability of these data, which were used under license for the current study, and so are not publicly available. Data are however available from the authors upon reasonable request and with permission of the InSCI Study Group.

## Abbreviations

SCI: Spinal Cord Injury
InSCI: International Spinal Cord Injury Survey
LHS-SCI: International Learning Health System for Spinal Cord Injury Study
WHO: World Health Organization
ISPRM: International Society for Physical and Rehabilitation Medicine
ISCoS: International Spinal Cord Society
PRM: Physical and Rehabilitation Medicine
GP: General Practitioner
