# Supplementary material for: Healthcare service utilization patterns and patient experience in persons with spinal cord injury: a comparison across 22 countries
Source: BMC Health Serv Res. 2022 Jun 7;22:755. doi: 10.1186/s12913-022-07844-3 (PMC9175375; doi:10.1186/s12913-022-07844-3)
Supplement: Supplementary file 1 — Additional file 1: Supplementary Table 1. InSCI study design characteristics by country. [file 12913_2022_7844_MOESM1_ESM.docx]

| **Country** | AU^b,c^ | BR^b,c^ | CN^b,c^ | FR^b,c^ | DE^b,c^ | GR^b,c^ | ID^b,c^ | IT^b,c^ | JP^b,c^ | LT^b,c^ | MY^b,c^ | MA^b,c^ | NL^b,c^ | NO^b,c^ | PL^b,c^ | RO^b,c^ | ZA^b,c^ | KR^b,c^ | ES^b,c^ | CH^b,c^ | TH^b,c^ | US^b,c^ |  |
| --- | --- | --- | --- | --- | --- | --- | --- | --- | --- | --- | --- | --- | --- | --- | --- | --- | --- | --- | --- | --- | --- | --- | --- |
| **Sample size, N (%)** | 1578 (12.5) | 201  (1.6) | 1354 (10.8) | 413  (3.3) | 1617 (12.9) | 200  (1.6) | 201  (1.6) | 206  (1.6) | 302  (2.4) | 218  (1.7) | 298  (2.4) | 385  (3.1) | 260  (2.1) | 610  (4.9) | 971  (7.7) | 216  (1.7) | 199  (1.6) | 889  (7.1) | 417  (3.3) | 1530 (12.2) | 320  (2.5) | 203  (1.6) |  |
| **Sampling frame** | | | | | | | | | | | | | | | | | | | | | | | |
| Predefined frame | x |  | x |  | x |  |  |  |  |  |  |  | x | x | x |  | x |  |  | x |  |  |  |
| Convenience sample |  | x |  | x |  | x | x | x | x | x | x | x |  |  |  | x |  | x | x |  | x | x |  |
|  | | | | | | | | | | | | | | | | | | | | | | | |
| **Place of recruitment** | | | | | | | | | | | | | | | | | | | | | | | |
| General / acute hospital |  |  | 29 |  |  | 2 | 9 |  | 3 |  | 7 | 6 |  |  | 1 | 2 | 3 |  | 4 |  | 3 | 2 |  |
| Rehabilitation institution | 6 | 2 |  | 1 | 8 | 2 |  | 4 |  | 1 | 1 | 8 | 3 | 3 | 3 | 2 |  | 1 |  | 4 | 1 | 2 |  |
| Patient organization | 3 |  |  |  |  | 3 | 4 | 7 | 1 | 1 | 1 | 3 |  |  | 1 | 1 | 1 | 1 |  | 2 |  | 2 |  |
| Governmental institution | 1 |  |  |  |  |  | 1 |  |  |  |  | 3 |  |  |  |  |  |  |  |  |  |  |  |
| Research database |  |  |  | 7 |  |  |  |  |  |  |  |  |  |  |  |  | 1 |  |  |  |  |  |  |
|  | | | | | | | | | | | | | | | | | | | | | | | |
| **Administration mode** | | | | | | | | | | | | | | | | | | | | | | | |
| Paper-pencil | x | x |  | x | x | x |  | x | x | x | x | x | x | x | x | x | x | x | x | x | x | x |  |
| Online | x |  | x | x | x | x | x | x |  | x |  |  | x | x | x | x |  | x |  | x |  | x |  |
| Smartphone |  |  | x |  |  |  |  |  |  |  |  |  |  | x |  |  |  |  |  |  |  | x |  |
| Face-to-face interview |  | x | x |  |  | x | x | x |  | x | x | x |  |  | x | x | x | x | x | x | x |  |  |
| Telephone interview | x | x | x |  |  |  |  |  |  |  |  | x |  | x | x |  |  |  |  | x |  | x |  |

**Supplementary Table 1.** **InSCI study design characteristics by country**^a^

^a^ Fekete C, Brach M, Ehrmann C, Post M, Middleton J, Battistella L, et al. Cohort profile of the International Spinal Cord Injury Community Survey implemented in 22 countries. Archives of Physical Medicine and Rehabilitation. 2020; 101(12):2103-2111.

^b^ AU – Australia, BR – Brazil, CN – China, FR – France, DE – Germany, GR – Greece, ID – Indonesia, IT – Italy, JP – Japan, LT – Lithuania, MY – Malaysia, MA – Morocco, NL – the Netherlands, NO – Norway, PL – Poland, RO – Romania, ZA – South Africa, KR – South Korea, ES – Spain, CH – Switzerland, TH – Thailand, US – the United States

^c^ Information is organized in the following order per country: 1. National study coordinating center. 2. Place of recruitment: hospitals, databases, patient organizations, etc. 3. The region where the sampling was taking place

AU: 1. John Walsh Centre for Rehabilitation Research, Sydney Medical School, Northern Clinical School; The University of Sydney, Sydney Australia. 2. Metro, rural and regional databases, Spinal Centres in four Australian states, Consumer organizations in two Australian states, Insurers, and policymakers. 3. New South Wales, Queensland, South Australia, Victoria.

BR: 1. The University of São Paulo, São Paulo. 2. University Hospital database (n = 1). 3. São Paulo State

CN: 1. Nanjing Medical University, Nanjing. 2. Hospital-based sampling (n = 29). 3. Jiangsu and Sichuan provinces

FR: 1. St Jacques University Hospital of Nantes, Nantes, France. 2. Hospital-based sampling (n = 1). 3. Western France

DE: 1. Hannover Medical School, Hannover. 2. Databases of SCI specialized centers. 3. Berlin, Bad Tennstedt, Bad Berka, Bayreuth, Hessisch Lichtenau, Heidelberg, Bad Wildbad, Ulm.

GR: 1. PRM Department General Hospital “G. Gennimatas”, Athens. 2. Rehabilitation departments of hospitals and centers (public and private sector), Associations of persons with SCI, Sports associations of persons with disabilities. 3. Athens, Patras, Florina, Patient organizations serving different cities in Greece.

ID: Medicine and Rehabilitation Department, Dr. Cipto Mangunkusumo National General Hospital, University of Indonesia, Jakarta. 2. Hospital and patient organizations-based sampling, Disability Residence Center in Jakarta (Sasana Bina Daksa Budi Bhakti, Wisma Chesire). 3. Jakarta

IT: 1. San Giovanni Battista Hospital, Foligno, Perugia, Italy. 2. Spinal Unit / Specialized Rehab centers (n = 4), Disability associations (n = 6; FAIP (National database), Associazione Paraplegici Lombardia (Regional database), Ancora ONLUS (Novara), RuotaLibera (Milano), Associazione IDEA ONLUS (Alessandria), B-Free (Torino). 3. Novara, Bergamo, Catania, Perugia

JP: 1. Fujita Health University, Toyoake. 2. The Japanese Association of Rehabilitation Medicine (JARM), Japan Medical Society of Spinal Cord Lesion Multicenter study, Wakayama Medical University (WMU), Kibikogen Rehabilitation Center for Employment Injuries, Yokohama City University. 3. Wakayama, Okayama, Kanagawa Perfectures

LT: 1. Vilnius university hospital Santariskiu klinikos, Vilnius. 2. Database from specialized rehabilitation centers: Vilnius University Hospital Santaros Klinikos, Palanga Rehabilitation Hospital; Lithuanian Association of Paraplegics; Insurance fund database. 3. Vilnius, Palanga

MY: 1. University of Malaya Medical Centre. 2. SCI rehabilitation service providers (n = 8) that represent different regions: University of Malaya Medical Centre, Kuala Lumpur, Cheras Rehabilitation Centre, Kuala Lumpur, Hospital Sungai Buloh, Selangor, Hospital Permaisuri Bainun, Ipoh, Perak, Hospital Pulau Pinang, Penang, Hospital Sultan Ismail, Johor Bahru, Johor, Hospital Queen Elizabeth, Sabah, Hospital Umum Sarawak. 3. Kuala Lumpur, Selangor, Perak, Penang, Johor, Sabah, Sarawak

MA: 1. Department of Rehabilitation Medicine, Clinical Neuroscience Laboratory, Faculty of Medicine, Mohammed Ben Abdallah University, Fez. 2. Rehabilitation facilities: University Hospital of Fez, Regional Hospital of Fez, Military Hospital of Rabat, University Hospital of Rabat, University Hospital of Oujda, University Hospital of Marrakech, Regional Hospital of Agadir, NOR Private Hospital Center of Casablanca; Acute & general hospitals: University Hospital of Fez, Regional Hospital of Fez, University Hospital of Oujda, Regional Hospital of Oujda, University Hospital of Rabat, University Hospital of Marrakech; Patient organizations: Mohammed VI National Center for People with Disabilities, SALMI semi-private Hospital center of Casablanca, Inclusion and Disability Association; Governmental agencies: Ministry of Health, Ministry of Family, Solidarity, Equality and Social Development, National Agency of Health Insurance. 3. Fez, Rabat, Oujda, Marrakech, Agadir, Casablanca

NL: 1. University Medical Center Groningen, University of Groningen. 2. Three rehabilitation centers with a specialization in SCI rehabilitation: Rijndam Rehabilitation, Rotterdam, De Hoogstraat Rehabilitation, Utrecht, Center for Rehabilitation, University Medical Center Groningen. 3. Rotterdam, Utrecht, Groningen

NO: 1. University of Oslo, Oslo. 2. SCI units: St. Olavs Hospital, Helse Bergen, Sunnaas Sykehus.

PL: 1. The Medical University of Warsaw, Medical University of Łódź. 2. Multiple sampling frames consisting of DPO database (Foundation of Active Rehabilitation), hospital 1 database (Mazovian Rehabilitation Center STOCER), hospital 2 database (Upper Silesian Rehabilitation Center REPTY), hospital 3 database (Health Centre of Brothers Hospitallers of St. John of God Poznan University of Physical Education Foundation of Active Rehabilitation.

RO: 1. Rehabilitation Hospital Felix Spa, Băile-Felix. 2. Data Bases: Rehabilitation Hospital Felix Spa, Motivation Romania, Elias University Hospital, Sibiu County University Hospital, Craiova County Hospital. 3. Oradea, Bucharest, Sibiu

ZA: 1. Stellenbosch University, Cape Town. 2. Sampling frame from inception academic database including level I hospitals (in the Western Cape Province) plus database kept of a specialized rehabilitation center in Gauteng Province. 3. Western Cape and Gauteng Province

KR: 1. National Rehabilitation Center and Hospital, Seoul. 2. 3000 NRC SCI patient database, 2500 KSCIA member database, and the 2000 Korea Society for Industrial Disaster Victim member database.

ES: 1. Universitat d’Andorra, Spain. 2. Hospital Universitari Vall d’Hebron (Barcelona), Hospital Universitario Virgen del Rocío (Sevilla), Hospital Universitario Insular Materno-Infantil de Gran Canaria (Canarias), HU Cruces Bilbao (Bilbao). 3. Bilbao, Barcelona, Sevilla, Canarias

CH: 1. Swiss Paraplegic Research, Nottwil. 2. Specialized SCI collaboration centers: Swiss Paraplegic Zentrum, Rehab Basel, CRR Sion, Bagrist Zürich; University hospital HUG Geneva; ParaHelp (home care institution); patient organization (Swiss Paraplegic Association).

TH: 1. Chiang Mai University, Chiang Mai. 2. Multiple sampling frames: 4 hospitals/facilities including 2 university hospitals and 1 A-level hospital, 1 National Rehab Institute. 3. Chiang Mai, Bangkok, Ratchaburi, Nonthaburi.

US: 1. University of Vermont, Burlington, Vermont. 2. Contacts of Kennedy Krieger (National), Listserv of Northeast Disabled Athletic Association (Vermont, New England), Listserv of Northeast Passage (New Hamshire), Social media of Kelly Bush Foundation (Vermont, New England), Special Interest group (Pennsburg, PA), UVM Medical Center Clinics, Extra-database: Social network. 3. Vermont, New England, Referral from multiple states
