# Supplementary material for: Healthcare service utilization patterns and patient experience in persons with spinal cord injury: a comparison across 22 countries
Source: BMC Health Serv Res. 2022 Jun 7;22:755. doi: 10.1186/s12913-022-07844-3 (PMC9175375; doi:10.1186/s12913-022-07844-3)
Supplement: Supplementary file 2 — Additional file 2: Supplementary Table 2. Characteristics of the healthcare systems of InSCI countries. [file 12913_2022_7844_MOESM2_ESM.docx]

**Supplementary Table 2. Characteristics of the healthcare systems of InSCI countries**

| **Country** | AU^a^ | BR^a^ | CN^a^ | FR^a^ | DE^a^ | GR^a^ | ID^a^ | IT^a^ | JP^a^ | LT^a^ | MY^a^ | MA^a^ | NL^a^ | NO^a^ | PL^a^ | RO^a^ | ZA^a^ | KR^a^ | ES^a^ | CH^a^ | TH^a^ | US^a^ |
| --- | --- | --- | --- | --- | --- | --- | --- | --- | --- | --- | --- | --- | --- | --- | --- | --- | --- | --- | --- | --- | --- | --- |
| **Healthcare system type**^b^ | | | | | | | | | | | | | | | | | | | | | | |
| National | x | x |  |  |  | x | mix | x |  |  | x | mix |  | x |  |  | mix |  | x |  | mix |  |
| Social |  |  | x | x | x |  |  |  | x | x |  |  |  |  | x | x |  | x |  |  |  |  |
| Private |  |  |  |  |  |  |  |  |  |  |  |  | x |  |  |  |  |  |  | x |  | x |
|  | | | | | | | | | | | | | | | | | | | | | | |
| **Centralized / decentralized system**^c^ | | | | | | | | | | | | | | | | | | | | | | |
| Centralized |  | x | x | mix |  | x |  |  | mix | x | mix | x | x |  | mix | x | mix | x |  |  | mix |  |
| Decentralized | x |  |  |  | x |  | x | x |  |  |  |  |  | x |  |  |  |  | x | x |  | x |
|  | | | | | | | | | | | | | | | | | | | | | | |
| **Primary care strength**^d^ | | | | | | | | | | | | | | | | | | | | | | |
| Strong | x |  |  |  |  |  |  |  |  | x |  |  | x |  |  |  |  |  | x |  |  |  |
| Medium / Weak |  | x | x | x | x | x | x | x | x |  | x | x |  | x | x | x | x | x |  | x | x | x |
|  | | | | | | | | | | | | | | | | | | | | | | |
| **UHC index**^e^ | 87 | 75 | 82 | 84 | 86 | 78 | 59 | 83 | 85 | 70 | 76 | 73 | 86 | 86 | 74 | 71 | 67 | 87 | 86 | 87 | 83 | 83 |
|  | | | | | | | | | | | | | | | | | | | | | | |
| **Population with household health expenditures > 10% of total household expenditure or income**^f^ | 2.5 | 11.8 | 24.0 | - | 1.5 | 16.9 | 4.5 | 9.3 | 10.5 | 12.9 | 1.5 | 20.5 | - | 5.1 | 14.1 | 13.4 | 1.0 | 12.0 | 7.9 | - | 1.9 | 4.3 |

^a^ AU – Australia, BR – Brazil, CN – China, FR – France, DE – Germany, GR – Greece, ID – Indonesia, IT – Italy, JP – Japan, LT – Lithuania, MY – Malaysia, MA – Morocco, NL – the Netherlands, NO – Norway, PL – Poland, RO – Romania, ZA – South Africa, KR – South Korea, ES – Spain, CH – Switzerland, TH – Thailand, US – the United States

^b^ Böhm K, Schmid A, Götze R, Landwehr C, and Rothgang H. Five types of OECD healthcare systems: empirical results of a deductive classification. Health policy. 2013;113(3):258-269.

WHO, European Observatory on Health Systems and Policies Health in Transition Country Profiles (HIT)

Common Wealth International Health Care System Profiles

WHO Health System Profiles

Belabbes S. The truth about health in Morocco: no health without workforce development. Wilson Center. 2020.

Ruger J, Kress D. Health financing and insurance reform in Morocco. Health Affairs. 2007;26(4):1009-1016.

Semlali H. The Morocco country case study: positive practice environments. World Health Organization. 2010; 22.

Zwarenstein M. The structure of South Africa's health service. African Health. 1994;(Spec No):3-4.

^c^ "The main characteristic of a decentralised government is the existence of several governing bodies, which have the power for political, administrative, or budgetary decisionmaking at a regional or local level. Three levels of government are defined: central/federal, state/province/region, and local/municipality. Different types of decentralisation include fiscal decentralisation (the transfer of financial resources in the form of grants and tax raising powers to sub-national units of government); administrative decentralisation (the functions of central government are shifted to geographically distinct administrative units); and political decentralisation (where powers and responsibilities are devolved to elected sub-national governments). The spending autonomy concept encompasses some facet of all these types of decentralisation, but mainly focuses on administrative decentralisation….. Accurately comparing and measuring decentralisation across countries is difficult." Beazley I, et al. Decentralisation and performance measurement systems in health care. OECD Working Papers on Fiscal Federalism. OECD Publishing, Paris. 2019; 28.

Sreeramareddy C, Sathyanarayana T. Decentralised versus centralised governance of health services. The Cochrane Database of Systematic Reviews. 2019(9).

WHO European Observatory on Health Systems and Policies Health in Transition Country Profiles (HIT)

Common Wealth International Health Care System Profiles

WHO Health System Profiles

Tejativaddhana P, Briggs D, Singhadej O, Hinoguin R. Developing primary health care in Thailand: Innovation in the use of socio-economic determinants, Sustainable Development Goals, and the district health strategy. Public Administration and Policy. 2018;21(1):36-49.

Soldi R, Odone C. The management of health systems in the EU Member States: the role of local and regional authorities. European Committee of the Regions. 2017.

^d^ Kringos D, Boerma W, Bourgueil Y, Cartier T, Dedeu T, Hasvold T, Hutchinson A, Lember M, Oleszczyk M, Pavlic D, Svab I. The strength of primary care in Europe: an international comparative study. British Journal of General Practice. 2013;63(616):e742-e750.

WHO European Observatory on Health Systems and Policies Health in Transition Country Profiles (HIT)

WHO Health System Profiles

Common Wealth International Health Care System Profiles

Pavlič D, Sever M, Klemenc-Ketiš Z, et al. Strength of primary care service delivery: a comparative study of European countries, Australia, New Zealand, and Canada. Primary Health Care Research and Development. 2018;19(3):277-287.

Tejativaddhana P, Briggs D, Singhadej O, Hinoguin R. Developing primary health care in Thailand: Innovation in the use of socio-economic determinants, Sustainable Development Goals, and the district health strategy. Public Administration and Policy. 2018;21(1):36-49.

Pongpirul K, Starfield B, Srivanichakorn S, et al. Policy characteristics facilitating primary health care in Thailand: A pilot study in the transitional country. International Journal for Equity in Health. 2009; 8.

World Bank Group. Is Indonesia ready to serve? An analysis of Indonesia’s primary health care supply-side readiness. 2018.

OECD. Primary health care in Brazil. OECD Reviews of Health Systems, OECD Publishing, Paris. 2021.

#### Primary health care in China. Editorial. The Lancet Regional Health, Western Pacific, 2020;3,100019.

Kato D, Ryu H, Matsumoto T, Abe K, Kaneko M, Ko M, Irving G, Ramsay R, Kondo M. Building primary care in Japan: literature review. Journal of General and Family Medicine. 2019; 20(5),170-179.

Belabbes S. The truth about health in Morocco: no health without workforce development. Wilson Center. 2020.

Ruger J. and Kress D. Health financing and insurance reform in Morocco. Health Affairs. 2007;26(4):1009-1016.

Semlali H. The Morocco country case study: positive practice environments. World Health Organization. 2010; 22.

^e^ UHC Index of Service Coverage: "coverage of essential health services (defined as the average coverage of essential services based on tracer interventions that include reproductive, maternal, newborn and child health, infectious diseases, non-communicable diseases and service capacity and access, among the general and the most disadvantaged population). The indicator is an index reported on a unitless scale of 0 to 100, which is computed as the geometric mean of 14 tracer indicators of health service coverage. The tracer indicators are as follows, organized by four components of service coverage: 1. Reproductive, maternal, newborn and child health 2. Infectious diseases 3. Noncommunicable diseases 4. Service capacity and access ". Data from WHO The Global Health Observatory, UHC Index of Service Coverage. (World Health Organization. UHC Index of Service Coverage: World Health Organization The Global Health Observatory. World Health Organization. https://www.who.int/data/gho/data/indicators/indicator-details/GHO/uhc-index-of-service-coverage. Accessed 27 Feb 2022.)

^f^ Data from WHO The Global Health Observatory, Population with household health expenditures greater than 10% of total household expenditure or income. (World Health Organization. Population with household health expenditures greater than 10% of total household expenditure or income. World Health Organization. https://www.who.int/data/gho/indicator-metadata-registry/imr-details/4836. Accessed 27 Feb 2022.)
