# Supplementary material for: Healthcare service utilization patterns and patient experience in persons with spinal cord injury: a comparison across 22 countries
Source: BMC Health Serv Res. 2022 Jun 7;22:755. doi: 10.1186/s12913-022-07844-3 (PMC9175375; doi:10.1186/s12913-022-07844-3)
Supplement: Supplementary file 3 — Additional file 3: Supplementary Table 3. Association between healthcare utilization type and patient experience. [file 12913_2022_7844_MOESM3_ESM.docx]

**Supplementary Table 3.** **Association between healthcare utilization type and patient experience**

|  |  | | **Unadjusted**^a^  (N = 9818) | |  | **Adjusted**^b^  (N = 9423) | |
| --- | --- | --- | --- | --- | --- | --- | --- |
|  |  | | Coeff | 95% CI, Sign^c^ |  | Coeff | 95% CI, Sign^c^ |
| *No visits to any provider* | |  | 0.5 | (-0.9, 1.9) |  | 0.5 | (-0.9, 1.9) |
|  | |  |  |  |  |  |  |
| *Healthcare provider visits* | |  |  |  |  |  |  |
| Primary care physician / GP | |  | 0.6 | (-0.4, 1.6) |  | 0.6 | (-0.4, 1.6) |
| PRM^d^ / SCI physician | |  | 2.1 | (1.2, 3.0)*** |  | 2.1 | (1.2, 3.0)*** |
| Other specialist physicians | |  | 0.0 | (-0.8, 0.9) |  | 0.0 | (-0.9, 0.9) |
| Nurse of midwife | |  | -1.5 | (-2.5, -0.6)** |  | -1.5 | (-2.5, -0.5)** |
| Dentist | |  | 1.1 | (0.2, 2.1)* |  | 1.1 | (0.1, 2.1)* |
| Physiotherapist | |  | 0.7 | (-0.3, 1.6) |  | 0.7 | (-0.2, 1.7) |
| Chiropractor | |  | -1.1 | (-3.4, 1.1) |  | -1.2 | (-3.5, 1.1) |
| Occupational therapist | |  | 0.8 | (-0.3, 2.0) |  | 1.0 | (-0.2, 2.1) |
| Psychologist | |  | -1.8 | (-3.2, -0.3)* |  | -1.6 | (-3.1, -0.1)* |
| Alternative medicine | |  | -1.4 | (-2.8, 0.0)* |  | -1.6 | (-3.0, -0.2)* |
| Pharmacist | |  | 0.7 | (-0.2, 1.7) |  | 0.7 | (-0.3, 1.7) |
| Home healthcare worker | |  | -2.6 | (-3.7, -1.5)*** |  | -2.3 | (-3.5, -1.2)*** |
| *Number of inpatient stays* | |  | 0.0 | (-0.0; -0.0) |  | 0.0 | (-0.0; -0.0) |
|  |  | |  |  |  |  |  |
| *Socio-demographic characteristics* | | | | |  |  |  |
| Female |  | |  |  |  | -0.2 | (-1.1, 0.7) |
| Age, years | | |  |  |  |  |  |
| 18–30 |  | |  |  |  | Ref. |  |
| 31–45 |  | |  |  |  | 0.0 | (-1.4, 1.3) |
| 46–60 |  | |  |  |  | 0.3 | (-1.0, 1.7) |
| 61–75 |  | |  |  |  | 0.7 | (-0.8, 2.2) |
| ≥76 |  | |  |  |  | -0.2 | (-2.4, 1.9) |
| No migrant background | | | | |  | 0.6 | (-1.1, 2.3) |
|  |  | |  |  |  |  |  |
| *SCI characteristics* | | | |  |  |  |  |
| Tetraplegia | | |  |  |  | -1.1 | (-1.9, -0.3)** |
| Incomplete lesion | | | |  |  | 1.0 | (0.2, 1.9)* |
| Nontraumatic etiology | | | | |  | 0.2 | (-0.9, 1.2) |
| Years lived with SCI | | | |  |  |  |  |
| <1 |  | |  |  |  | Ref. |  |
| 1–5 |  | |  |  |  | -1.4 | (-5.0, 2.2) |
| 6–10 |  | |  |  |  | -0.6 | (-4.3, 3.1) |
| 11–15 |  | |  |  |  | -0.1 | (-3.8, 3.6) |
| 16–20 |  | |  |  |  | -1.7 | (-5.6, 2.1) |
| 21–25 |  | |  |  |  | 0.1 | (-3.8, 4.1) |
| 26–30 |  | |  |  |  | -0.8 | (-4.8, 3.3) |
| 31–35 |  | |  |  |  | -0.6 | (-4.7, 3.6) |
| 36–40 |  | |  |  |  | -1.4 | (-5.9, 3.0) |
| ≥41 |  | |  |  |  | -2.1 | (-6.1, 1.9) |
|  |  | |  |  |  |  |  |
| *Constant* |  | | 62.5 | (58.8, 66.3)*** |  | 62.3 | (56.9, 67.9)*** |
| Regression analysis of health care utilization, socio-demographic and SCI characteristics on healthcare experience score obtained after Rasch analysis (0-100)  ^a^ Country level variance: 18%, chi-square = 1563.1, p-value < 0.001  ^b^ Country level variance: 18%, chi-square = 1386.3, p-value < 0.001  ^c^ * p < 0.05 ** p < 0.01 *** p < 0.001  ^d^ PRM – physical and rehabilitation medicine | | | | | | | |
