# Supplementary material for: Healthcare service utilization patterns and patient experience in persons with spinal cord injury: a comparison across 22 countries
Source: BMC Health Serv Res. 2022 Jun 7;22:755. doi: 10.1186/s12913-022-07844-3 (PMC9175375; doi:10.1186/s12913-022-07844-3)
Supplement: Supplementary file 4 — Additional file 4: Supplementary Table 4. Association between healthcare utilization pattern and patient experience. [file 12913_2022_7844_MOESM4_ESM.docx]

**Supplementary Table 4.** **Association between healthcare utilization pattern and patient experience**

|  |  | **Unadjusted**  **(N = 12588)** | |  | **Adjusted**  **(N = 11838)** | |
| --- | --- | --- | --- | --- | --- | --- |
|  |  | Coeff | 95% CI, Sign^a^ |  | Coeff | 95% CI, Sign^a^ |
| Cluster 1 |  | Ref. |  |  | Ref. |  |
| Cluster 2 |  | -20.6 | (-21.9, -19.2)*** |  | -20.2 | (-21.6, -18.8)*** |
| Cluster 3 |  | -5.3 | (-6.4, -4.3)*** |  | -5.5 | (-6.6, -4.3)*** |
| Cluster 4 |  | -17.4 | (-18.7, -16.4)*** |  | -16.4 | (-17.8, -15.0)*** |
| Cluster 5 |  | -9.8 | (-11.3, -8.4)*** |  | -9.5 | (-11.0, -8.0)*** |
| Cluster 6 |  | -14.3 | (-15.7, -12.9)*** |  | -13.6 | (-15.1, -12.2)*** |
| Cluster 7 |  | 2.1 | (-0.4, 4.5) |  | 3.9 | (1.3, 6.4)** |
| Cluster 8 |  | -1.9 | (-3.5, -0.2) |  | -0.5 | (-2.2, 1.3) |
| Cluster 9 |  | -12.6 | (-15.5, -9.6)*** |  | -11.0 | (-14.0, -8.0)*** |
|  |  |  |  |  |  |  |
| *Socio-demographic characteristics* | | | |  |  |  |
| Female |  |  |  |  | -0.1 | (-0.9, 0.8) |
| Age, years | |  |  |  |  |  |
| 18–30 |  |  |  |  | Ref. |  |
| 31–45 |  |  |  |  | 1.1 | (-0.3, 2.4) |
| 46–60 |  |  |  |  | 2.2 | (0.9, 3.5)** |
| 61–75 |  |  |  |  | 3.9 | (2.5, 5.3)*** |
| ≥76 |  |  |  |  | 2.3 | (0.4, 4.2)* |
| No migrant background | | | |  | -0.1 | (-1.5, 1.3) |
|  |  |  |  |  |  |  |
| *SCI characteristics* | | |  |  |  |  |
| Tetraplegia | |  |  |  | -1.2 | (-1.9, -0.4)** |
| Incomplete lesion | | |  |  | 1.6 | (0.8, 2.4)*** |
| Nontraumatic etiology | | | |  | 0.2 | (-0.7, 1.2) |
| Years lived with SCI | | |  |  |  |  |
| <1 |  |  |  |  | Ref. |  |
| 1–5 |  |  |  |  | 0.8 | (-2.8, 4.4) |
| 6–10 |  |  |  |  | 2.4 | (-1.2, 6.0) |
| 11–15 |  |  |  |  | 3.4 | (-0.3, 7.1) |
| 16–20 |  |  |  |  | 2.1 | (-1.6, 5.8) |
| 21–25 |  |  |  |  | 3.3 | (-0.5, 7.1) |
| 26–30 |  |  |  |  | 2.3 | (-1.5, 6.2) |
| 31–35 |  |  |  |  | 3.0 | (-1.0, 7.0) |
| 36–40 |  |  |  |  | 3.7 | (-0.5, 7.8) |
| ≥41 |  |  |  |  | 1.9 | (-2.0, 5.8) |
|  |  |  |  |  |  |  |
| *Constant* |  | 72.3 | (71.4, 73.2)*** |  | 67.3 | (63.3, 71.3)*** |
| Regression analysis of health care utilization clusters on healthcare experience score obtained after Rasch analysis (0-100), adjusted for socio-demographic and SCI characteristics.  ^a^ * p < 0.05 ** p < 0.01 *** p < 0.001 | | | | | | |
